# Uptake of Health Care Services by Refugees: Modelling a Country Response to a Western Balkan Refugee Crisis

**DOI:** 10.3390/healthcare8040560

**Published:** 2020-12-14

**Authors:** Milena Santric-Milicevic, Milena Vasic, Vladimir Vasic, Mirjana Zivkovic-Sulovic, Dragana Cirovic, Milan Lackovic, Nikolina Boskovic

**Affiliations:** 1 Institute of Social Medicine, Faculty of Medicine, University of Belgrade, 11000 Belgrade, Serbia; 2 Institute of Public Health of Serbia, 11000 Belgrade, Serbia; milena_vasic@batut.org.rs (M.V.); mirjana_sulovic@batut.org.rs (M.Z.-S.); 3 Department of Statistics and Mathematics, Faculty of Economics, University of Belgrade, 11000 Belgrade, Serbia; vladimir.vasic@ekof.bg.ac.rs; 4 Faculty of Medicine, University of Belgrade, 11000 Belgrade, Serbia; dragana.cirovic@med.bg.ac.rs; 5 University Children’s Hospital, 11000 Belgrade, Serbia; 6 Clinical Hospital Center “Dr Dragiša Mišović”, 11000 Belgrade, Serbia; milan.lackovic@dragisamisovic.bg.ac.rs; 7 Fulbright Scholar at the Institute of Social Medicine, Faculty of Medicine, University of Belgrade, 11000 Belgrade, Serbia; boskovic@bu.edu

**Keywords:** refugees, crises intervention, needs assessment, delivery of health care, disaster planning, ARIMA Modelling

## Abstract

Planning and adjusting health capacities to meet the needs of refugees is a constant issue for transit and destination countries following the 2015/2016 Western Balkans refugee crisis. Understanding this crisis is important for taking the right steps in the future. The study informs about the prediction of the refugees’ health needs and demands for services in correspondence to political decision-making during 2015/2016 Western Balkan Refugee Crisis. Time series analysis, linear regression, and correlation analyses modelled the weekly flux of arrivals of more than half a million refugees to Serbia and the European Union, changes in the utilization of health care services, and disease diagnoses. With strategic planning, in the event of a recurrence of the refugee crises, the demand for health care services in the transit country could increase by 63 (95% CI: 21–105) for every additional 1000 refugees.

## 1. Introduction

As estimated by the United Nations, 60 million people worldwide are forcefully displaced from their homes for various reasons (i.e., war, violence, and economic instability). The majority of them are forced to cross several borders before finding adequate permanent settlements, whilst others are internally displaced within their own country. This paper focuses on the health needs and demands for health care services of the vulnerable population of refugees and asylum seekers (hereinafter refugees). This population is also named ‘mixed-migrants’, and the term encompasses migrants, refugees, and asylum-seekers who typically travel together using the same transportation and routes and who are seeking residence in other countries for longer than one year [1,2]. Undocumented migrants face major barriers to accessing health care services and other social support services [3]. Asylum-seekers and refugees have expressed rights to health care, social housing, and support for finding stable employment, and resources for integrating into society [4]. An effective response to unexpected crises, such as refugee crises, requires that transition countries adequately prepare in order to avoid disruption in the functioning of health, social and other public systems [3].

The huge 2015–2016 Western Balkan Refugee Crisis (hereinafter as WBRC) [5] was characterized by the several waves of arrivals of the refugee population from countries facing internal conflict to transit and destination countries. The pressure from the media in the countries on the Balkan route to European Union (EU), has framed the WBRC as an issue of should the borders remain open or be closed [6]. At the same time, in the countries of destination, attention has been paid to the capacity of the social protection and economic system for accommodation and employment of refugees upon admission from transit countries [7]. Uncertainty about the duration and complexity of travel to destination countries has shaped the ability of transit countries to meet the needs of refugees and provide adequate protection and care for the health of refugees while staying in the country. It also highlighted the dynamics of the humanitarian aid framework more relevant than ever before [8]. Since the population in Serbia has experienced similar refugee crises and internal displacement most recently during the 1990s when one million refugees were sheltered in 700 centers throughout the country [9], the WBRC created a compassionate atmosphere for refugees among the domicile population and the main issue in Serbia was how to adjust the health care capacities to effectively cover the needs of refugees who were trying to settle or transit the country [10].

Both transition and destination countries face challenges in coordinating, managing, and monitoring access to health care services to ensure the health of refugee population. Complications due to incomplete reliable data on their number, routes, health status, health risks, and habits make contingency planning of health service provisions for refugees difficult [11]. The absence of health care contingency plans for refugees makes adequately responding to the needs of this vulnerable population challenging [12]. This motive of study is to enable strategic planning for the provision of health care for refugees in both transit and destination countries in the future, by increasing the understanding the response of a transit country to WBRC. This was accomplished by describing and modelling characteristics of refugees’ arrivals and their uptake of health care services in Serbia, which was the transition country for refugees during the 2015–2016 WBRC [5]. The Autoregressive Integrated Moving Average—ARIMA models with outlier detection is one of the most accurate methods for the analysis of one-dimensional time series [13]. Using it, we can very accurately assess the movement of the analyzed time series, as well as to notice all the structural fractures that have occurred on the basis of external (political) influences [14,15].

The key political context during WBRC along with the frailty of political setting in the Western Balkans has been shaped by armed conflicts and the breakdown of ex-Yugoslavia at the beginning of nineties of the last century. After more than two decades hurtful remembrances still exist. The existing relations between Former Yugoslav Republic of Macedonia and Greece regarding the name of Macedonia, between Croatia and Slovenia due to unsolved border disagreement, different relations of WB countries with the EU and access to European funds to deal with WBRC could have meaningful implications on coping with the crisis.

Parliamentary Assembly of the Council of Europe specified six periods in the evolution of WBRC [5], and labeled them according to the significant events in each period: I—from June to August (Prior to August), II—between August and September 2015 (Chaos and Panic), III—between October and November 2015 (Stability and Order), IV—Between November 2015 and February 2016 (Restrictions and Confusion), V—from mid-February until March 2016 (Quotas and Tensions), and VI—in March (EU—Turkey Summit) [5]. In June 2015, North Macedonia revised its asylum law to allow refugees and migrants to enter the country legally (a similar approach to that already applicable in Serbia) which accelerated their transit time through the country. Prior to August 2015, the number of mixed-migrants ‘transits through the Serbia have increased exponentially, resulting with about 108,000 arrivals in Hungary in August 2015 [5]. From August to September 2015, chaos and panic were rife among the refugees and the general population due to tremendously huge arrivals of migrants reaching 3000 per day. Political decisions to close borders of North Macedonia and Hungary (with the wire fence) with Serbia led to blockade of the borders and intensification of protests. The migrant’s flow was directed to Croatian border and in two days almost 13,000 people entered Croatia, which instead of registration and accommodation to new arrivals, organized the transport of migrants towards Hungarian borders, but Hungary has also built the wire fence along the border with Croatia in October. The third period, the establishment of registration points for refugees, the access points to essential medical and social care, food, and water, and for a short-term break has brought “stability and order” on the Western Balkan route in October and November. However, the situation became temporarily complicated because of a wire fence at one part of the Croatian-Slovenian border. Also, despite criticism by the United Nations, since October 18th, North Macedonia, Serbia, Croatia, and Slovenia decided to decrease daily arrivals by admitting only “SIA nationalities” (Syrians, Iraqis, and Afghans). Austria and Slovenia have returned them to the countries of previous entrance. Those measures caused the protests of migrants and refugees at the border of Greece and North Macedonia followed by reaction of their police forces with use of batons, tear gas and rubber bullets. The key element of the restriction and confusion in the fourth period, from November 2015 to February 2016 was that a significant number of people were blocked in Greece since Greece didn’t apply nationality screening. However, till February 2016 the nationality screening didn’t have significant effects, countries alongside the Western Balkan route decided to introduce a new policy measure- to admit only citizens of Syria and Iraq with the identity cards with photographs. Austria accepted only 80 asylum seekers per day. In the fifth period on 26 of February, North Macedonia, Serbia, Croatia, and Slovenia restricted the daily admission of migrants and refugees and introduced quota at 580. The number of returns to the neighboring countries along the Balkan route has increased. The European Commission concerns were raised with the absence of the plan to reach the target of new 50,000 permanent reception capacity and this situation produced tensions between Greece, West Balkans countries and Austria. By the end of March, the Greece faced the risk of having at least 70,000 refugees and migrants trapped in the country requiring a shelter. In the sixth period, the EU—Turkey summit was held on 7 of March 2016, which resulted with a prompt implementation of the EU-Turkey Joint Action Plan to stem refugee flows and address trafficking and smuggling. The plan included Turkey taking back the refugees and migrants arriving from Greek islands asking in return additional financial assistance of 3 billion € in order to support Syrian refugees in the next three years, and fast-track for Turkish EU accession. The next day, Slovenia, Croatia, Serbia and FYRM, declared new measures aimed to block all transit migration. It resulted with the termination of the Balkan route as it was before. Given the dynamic and flow of political decisions to address the WBRC, our assumption was that the healthcare system of Serbia has been adapting accordingly.

The primary objective of the study was to identify how Serbia’s health care capacities adapted to meet the health needs of refugees and utilizing these results to provide evidence-based policy recommendations. The overall aim of the study was to model the uptake of health care services by refugees in terms of the contextualized political response to the 2015/2016 WBRC so that decision-makers in the transit country could be informed about the possible demand for health care services in the event of a recurrence of the refugee crises.

## 2. Materials and Methods

The study population encompassed refugees in Serbia between June 2015 and March 2016. The refugee challenge was described using two variables: the number of arrivals of refugees in Serbia and to EU countries (Croatia, Slovenia, Austria, and Hungary), and number of refugees registered by the Serbian health care providers. The capacity of the national health system for covering the health needs of this population was described using two variables, the total number of their medical conditions (e.g., infections, non-communicable diseases (NCDs), injuries, etc.) and the total number of health care services utilized (e.g., preventive, curative, diagnostic, and transport services). One assumption made was that the strain posed on the capacity of the national health system for covering essential health needs of refugees in Serbia. Data on refugee arrivals to EU and to Serbia are sourced from the United Nation Refugee Agency—UNHCR data portal [16]. The weekly data about health care services and medical conditions of refugees were obtained from the electronic database of the Institute of Public Health of Serbia (IPHS) [17], which the health care providers have originally collected. The study design, data and methodology were reviewed and approved by the Center for Health Care Analysis, Planning and Organization of the Institute of Public Health of Serbia, in March 2016. The number of refugees’ arrivals, their registration, their medical conditions, and their utilization of health care services during the variable political context of WBRC [5] were described on a weekly level from 1 June 2015 to 3 April 2016; in total 31 weeks where only the first week represents data from 1 June to 5 October 2015 [17].

The six periods of the WBRC that were explored in this study were labeled by the Parliamentary Assembly proposed of the Council of Europe [5]: 1 June–August (Prior to August period I), 5 September–4 October 2015 (Chaos and Panic period II), 5 October–22 November 2015 (Stability and Order period III), 23 November 2015–14 February 2016 (Restrictions and Confusion period IV), 15 February- 6 March 2016 (Quotas and Tensions period V), and 7 March–3 April (EU—Turkey Summit period VI) [5].

The weekly data of the study variables were presented as total number, mean, standard deviation, range, and the percent change from to stage to the stage of WBRC. Using the ARIMA models with outlier detection we wanted to detect direct influence of political decisions on the movement of time-series. Namely, the direct consequences of political decisions are reflected in the appearance of structural breaks in the movement of the time-series [18], and these breaks are detected as outliers in ARIMA modeling [13]. Any structural break in the time-series that occurred under the influence of an external (political) factor can be detected using ARIMA models with outlier detection procedure [19]. With any other modelling procedure, that would be very difficult, almost impossible [13]. Although ARIMA is strong forecasting tool, it produces plausible short-term forecasts [20], i.e., for the next two three weeks. Therefore, correlation analysis in this study provided evidence that could serve to predict future refugee crises [13].

ARIMA time-series analysis, including outlier analysis, were applied to explore the changes of study variables (estimates of total numbers with 95% Confidence Interval—CI), and affected by the political context of the WBRC evolution. Furthermore, the Spearman’s rho nonparametric correlation analysis and the linear regression analysis (*p* significance = 0.05) were applied to assess the relationship between the-number of arrivals of refugees in Serbia, the number of medical conditions, as well as the number of health care services utilized. Statistical analyses were done in IBM SPSS Statistics 25.

## 3. Results

This section describes the number of arrivals of refugees to Serbia and the EU during the WBRC evolution per week, their medical conditions, and health care services utilized. In additional texts, the estimates of the study variables reflecting the effect of political changes regarding WBRC were established with the ARIMA modelling of their time series, correlation, and linear regression.

The health care services were provided by trained professionals in 56 health institutions: 29 primary health care institutions, 20 hospitals and seven public health institutes. Services were also provided by seven domestic and international non-governmental organizations (NGOs) and one private clinic. The most commonly-provided services were curative examinations (93%), preventive examinations (3%), medical transport (1%), laboratory services (2%), and medical transport (1%). In accordance with needs, refugees were referred to diagnostic procedures and further treatment to receive a higher level of health care in competent institutions.

While transiting Serbia, from 1 of June 2015 to 4 of April 2016, health care providers in Serbia recorded a total of 103,090 refugees, identified a total of 102,105 medical conditions and provided 108,397 health care services. In the period from 1 of October 2015 to 4 of April 2016, there were 527,001 arrivals of refugees to Serbia, and a total of 1,726,148 of refugees traveling further.

Each week, between August and September 2015 (Chaos and Panic phase), between 276 and 2583 refugees were registered in centers and shelters (Table 1), averaging 4.3% of weekly arrivals. On average, 1733 medical conditions were registered each week, mainly non-communicable diseases (NCDs), and respiratory and gastrointestinal infections. Of the total 1958 health care services utilized per week, most were curative (1655.6), diagnostic (306.6), and preventive screening services (31.8). Medical transport [20], and hospitalizations (9.4) averaged the least utilization for this and all periods of the crisis.

Between October and November (stability and order phase), between 1785 and 10,833 refugees were housed (Table 1), representing 31.1% of average weekly arrivals. Health care providers recorded 13.4% refugees on average in the country. Out of 4597.6 medical conditions registered each week, the majority were respiratory infections, NCDs, and gastrointestinal infections. The trend in the type of health care services used remained the same in this and all period of the study.

Between November and February (restrictions and confusion phase), there were between 2482 and 4474 refugees in shelters and centers (Table 1), representing 30.2% of weekly arrivals. By the end of 2015, health care providers recorded an average of 19.8% of all refugees, and 3578.6 different medical conditions, including respiratory infections (1913.7), NCDs (1110.9) and gastrointestinal infections (258.8).

The centres and sheltres in Serbia registered between 2249 and 3222 refugees each week from mid-February (Quoats and Tensions phase) until March (Table 1), accounting for 30.2% weekly arrivals. All refugees were registered by health care providers during this period. The refugees seeking asylum had 9.3 check-ups a week on average at this time.

In March (EU—Turkey Summit phase), between 1651 and 2563 refugees were accomodated (Table 1), averaging 5.6% of weekly arrivals in total. An average of 2105.0 medical conditions were registered. Among them, asylum-seeking refugees received 23.8 check-ups per week on average.

Stationary R squared parameters showed that all ARIMA models were statistically significant. The implicit and explicit specifications of the models are presented in Table 2 and can be used for predicting the study variables of interests if the scenario of similar flows of refugees and the dynamic of the political context repeat in the future.

**Figure 1:**
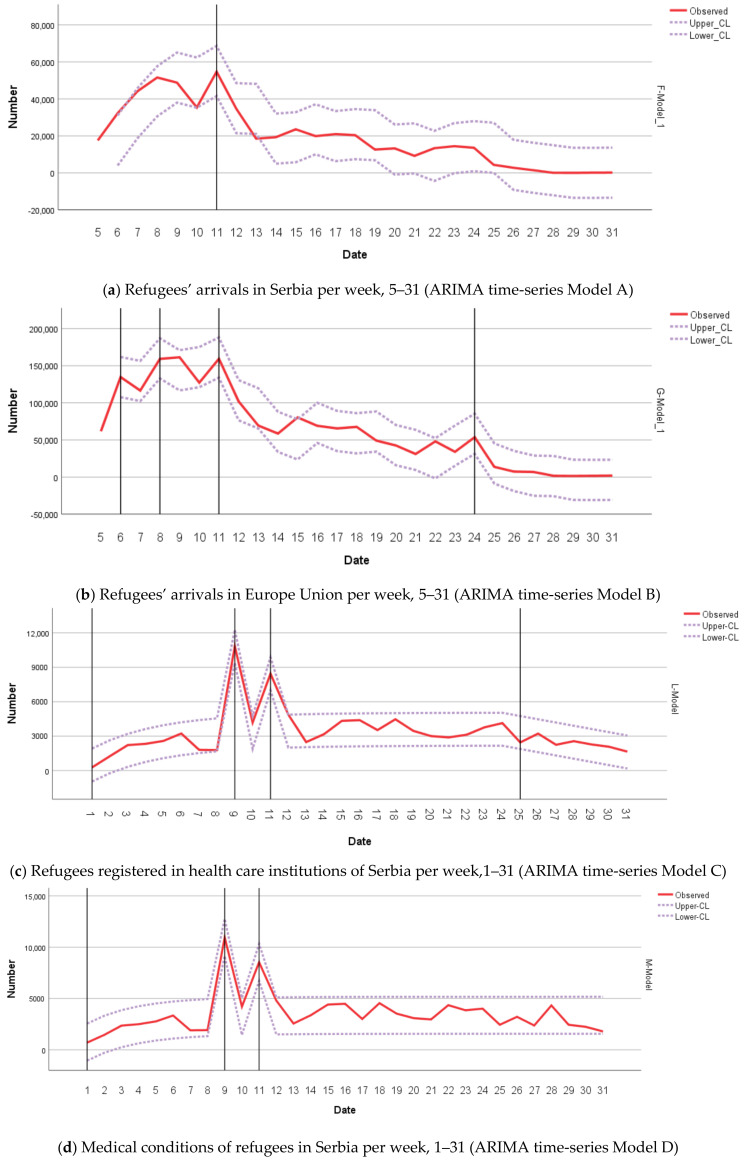

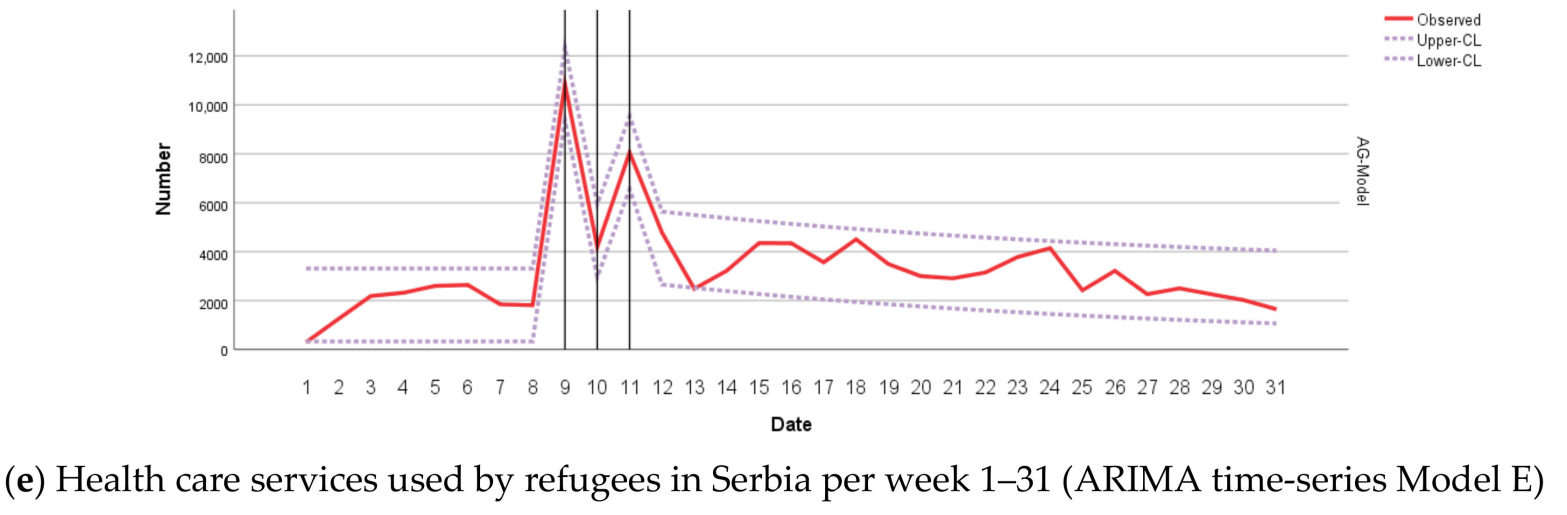
ARIMA time series models of Western Balkan Refugee Crises 2015/2016: (**a**) number of refugees’ arrivals in Serbia (Model A); (**b**) number of refugees’ arrivals in the Europe Union (Model B); (**c**) number of refugees registered in health care institutions of Serbia (Model C); (**d**) number of medical conditions of refugees in Serbia (Model D); and (**e**) number of health care services used by refugees in Serbia (Model E).

Mid-November (week 11), one additive outlier was observed in the ARIMA (0,1,0) model of the arrival number of refugees to Serbia. That outlier points to the short-term effect of political decisions made in that period, i.e., number of arrivals of refugees dropping by 36.8% and 46.4% in the subsequent two weeks (Figure 1a, Supplementary Table S1).

The ARIMA (0,1,0) model of number of arrivals of refugees to the EU had four outliers (Figure 1b, Supplementary Table S1). The innovative outlier in week 6 points to the long-term effects of political decisions made in that period, which manifested as increased weekly arrivals to the EU. With that tendency, the outlier in week 8 was a transient change with magnitude 4868.6 and decay factor of 79%. Two last additive outliers showed the short-term increases of arrivals in weeks 11 (by 25.3%) and 24 (by 57.8%).

There were four outliers in the ARIMA (0,0,0) model of numbers of refugees recorded by health care providers in Serbia (Figure 1c, Supplementary Table S1). In week 1, the transient outlier showed a low number of registered refugees with the magnitude of −3123.6 and decay factor of 77%. Two additive outliers in weeks 9 and 11 show a short-term increase in the total number of refugees by 506.9% and 102%, respectively recorded by health care providers. The local trend outlier in the week 25 points to the 40.7% decrease of refugees recorded by health care providers.

The ARIMA (0,0,0) model of the total number of refugees’ medical conditions had three outliers (Figure 1d, Supplementary Table S1). In weeks 9 and 11, the additive outliers showed 499.4% and 91.6%, respective increases of identified medical conditions. The transient outlier in the week 10, points to a decrease of identified medical conditions among refugees with magnitude of 2624.2 and decay factor of 94%.

There were three outliers in the ARIMA (0,0,0) model of the total number of health care services used (Figure 1e, Supplementary Table S1). In week 1, the transient outlier shows a low number of health care services used by refugees with magnitude of −2610.8 and decay factor of 71%. Two additive outliers, in the week 9 and 11, show short-term increases by 471.3% and 103% of health care services provided to refugees, respectively.

**Figure 2:**
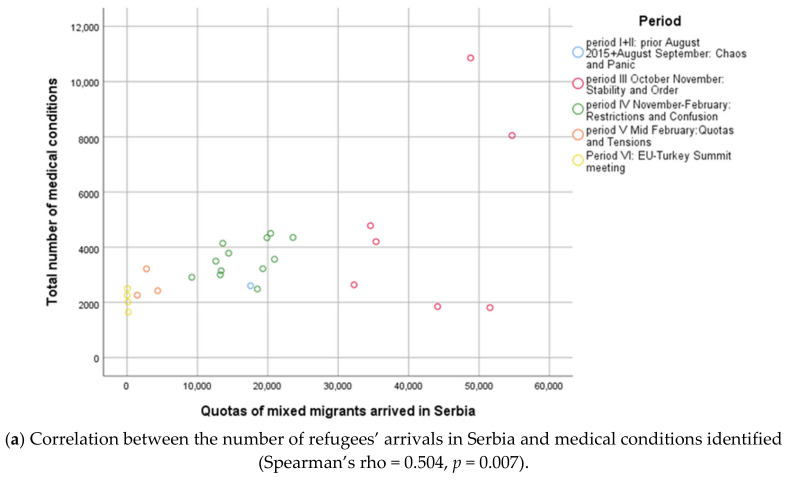

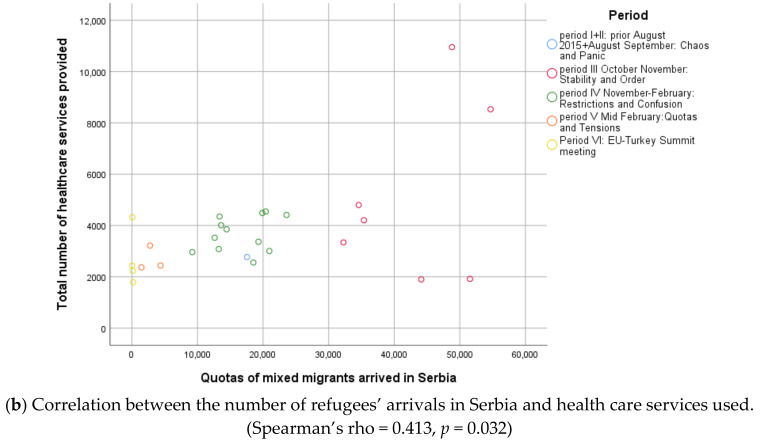
The number of refugees’ arrivals in Serbia, their medical conditions (**a**) and health care services utilisation (**b**) during the Western Balkan Refugee Crises 2015/2016.

Figure 2 presents the direct, positive, and significant correlation between the number of arrivals of refugees in Serbia and the number of medical conditions identified per week (Spearman’s rho = 0.504, *p* = 0.007), as well as the number of health care services used by refugees per week (Spearman’s rho = 0.413, *p* = 0.032). For each additional 1000 refugees in Serbia, there was an increase for 66 (95% CI: 25–107) in the average number of identified medical conditions, and an increase for 63 (95% CI: 21–105) in the average number of health care services used, that is almost one-third increase of health needs and services per week.

## 4. Discussion

The main study results base on the models of the week number of arrivals of refugees to Serbia and the EU, changes in utilization of health care services, and disease diagnoses. The models cover the ten months of the WBRC, and helped to identify how Serbia’s health care capacities adapted to meet the medical needs more than half a million refugees who transited Serbia. The Republic of Serbia, with significant assistance from international partners, managed the WBRC using the considerable experience in particular regarding the setting the legal and regulative conditions for protection of human rights related to the health care [16,17,21,22,23,24,25,26,27,28,29]. The decisions made during the WBRC were coordinated among the countries with the aim to allow them to deal with the crisis. The frequent changes of policies were result of insufficient effects of the previous ones, as well as the conflicted measures taken by neighboring countries [5].

Study results also that weekly, Serbia registered about 20,000 refugees and provided 1958 health care services (mostly curative, diagnostic and screening services) in total of 56 health care institutions. On average, during the WBRC, one refugee recorded by the Serbian health care provider had been diagnosed with one medical condition and used one health service on average. For each additional 1000 refugees, there were approximately one third increases in the average number of identified medical conditions and health care services used in a week. The majority of the treated conditions were communicable disease, while chronic NCDs were rarely diagnosed, which confirms a relatively good physical health of the refugees. This result was in accordance of previous studies findings, where infectious diseases were mostly reported as well as headeacks and injuries [3,30,31,32]. During the WBRC, Serbia put considerable efforts into addressing the health needs of the refugee population, who comprised 10% of the total domicile population. Despite the severe limitations of the national health budget imposed in 2014, it was widely accepted that refugees must not face barriers in accessing shelter and health care services [10]. The Serbian population was mostly accepting and supportive to the refugees transiting through Serbia toward EU countries. About 2.5 million people applied for asylum in the EU throughout the years of 2015 and 2016 [6]. Additionally, the EU was the largest contributor of emergency assistance to Serbia with nearly 80 million Euros for the improvement of border administration and provisions of health and education services, averaging approximately €160 per refugee since 2015 [33]. Although a study showed that in each stage of the WBRC, health care providers could have captured only 36.1% of all refugees in centers/shelters of Serbia and have registered just 15% of refugees that were entering the EU, it is important to stress that, during this period, Serbia did not face disease outbreaks and similar public health problems among refugees, and took all necessary steps to preserve and improve the health status of this vulnerable population.

Evidence from the study supports strategic planning for the provision of health care services in both transition and destination countries. If refugee crises repeat in the future, outputs of this study ARIMA models can be used for identifying the impact of political decisions on the flows of refugees, their health needs and demands for services. For instance, changes in the number of arrivals of refugees in Serbia in week 1 probably reflect the recording method which the health care providers were adjusting across the country [10,17], as well as irregularity of refugees’ flows and effects of the European Agenda on the Migration [34]. In June 2015, the Government of Serbia established an Inter-ministerial Working Group on Mixed Migration Flow to work on the national strategy for addressing the crisis [35]. In September 2015, Serbia’s National Assembly approved the Response Plan for addressing the massive influx of refugees. The plan identifies competent authorities, organizations and institutions, and their responsibilities in the event of a mass influx of refugees, as well as measures and activities to be undertaken and outlined necessary resources. The Ministry of Health approved the Plan for Health Protection of Migrants and Asylum Seekers in the Republic of Serbia that included health surveillance and provision of health care services [35].

In September (Supplementary Table S1), the Extraordinary Justice and Home Affairs Council [36] formally adopted a temporary and exceptional relocation scheme based on the emergency relocation proposal of the European Commission, and an agreement on military deployment in the Mediterranean and activation of hotspots in Italy and Greece. These political decisions brought slow change in the refugee arrivals to EU that has gradually continues forward through time—innovational outlier [13,37,38]. However, Hungary has closed the borders with Serbia causing a chaos and panic and activation a route through Serbia toward Croatia [5]. Therefore, at the European Council [39] and High-level Conference on the Eastern Mediterranean—Western Balkans route [40] the declaration was adopted and including the support to affected transit countries such as humanitarian assistance such as food, shelter, health care, psycho-social support, protection, water and sanitation, to refugees, asylum seekers and migrants transiting, enhancement of reception and accommodation facilities, capacity to manage borders and to ensure prompt registration of all refugees and migrants and effective, rapid and quality processing of asylum applications, awareness-raising and outreach activities at community level, and regional coordination and information exchange, among else [39]. The adoption and adherence to declaration monitored by the EU leaders [41] were political decisions with an impact that gradually disappeared, which were identified as transient outliers in the time-series of refugee arrivals to the EU, and of health care services provided to refugees (Supplementary Table S1). Similar impact had the European Agenda on Migration of the European Commission [34], and the advice the European Centre of Diseases Control regarding the health needs of the refugees [41], which Serbia as other Western Balkan countries followed to securitize a swift transit of refugees to the next country on the migration route [41,42]. That impact was identified as a transient outlier in the number of refugees registered in the health care institutions (Supplementary Table S1).

The sudden change, as additive outlier, was observed in the first period, after the implementation of several different and concrete measures of the European Commission to respond to the current migration challenges [34]. It was also seen at the end of the second period when the high-level meeting was held to increase operational cooperation all along the migration route in the Western Balkan [39]. On 25 October 2015, the Heads of State or Government of countries on the Western Balkan Refugee Route met and jointly agreed on a 17-point plan of pragmatic and operational measures including permanent exchange of information, limiting secondary movements, supporting refugees and providing shelter and rest, managing the migration flows together, border management, tackling smuggling and trafficking, information on the rights and obligations of refugees and migrants and monitoring on a weekly basis [39]. Those political decisions have brought stability and order in the refugee arrivals to Serbia, refugees’ registration in healthcare institutions, health care services use by the refugees, and medical conditions detected.

A short and local change of the trend, local trend outlier [13], was seen in the mean of the estimated number of refugees registration in the health care institutions, probably as a result of several political decisions taken to reduce the number of refugees in the EU, announcement of the closure of the Balkan route, and that North Macedonia, Serbia, Croatia and Slovenia will also reduce their numbers of refugees, and that Austria and Germany cap number of asylum applications per day plus persons allowed to transit per day [41].

The study results show that local health care providers increased the process of registration of refugees from about 5% to 100% throughout the evolution of the 2015–2016 WBRC. This meant that at the start of the crisis, data recording did not capture the total number of refugees and the health care services they used. Additionally, exploring Serbia’s health care intervention during the 2015–2016 WBRC as was done in this study, could be used to inform contingency planning for future refugee crises. Serbia has been putting considerable effort into better organizing health care provisions [17,35], but not all refugees necessarily utilized them [10]. Study results indicated that between each phase of the WBRC, the average number of health care services used steadily increased from about 2000 to almost 5000 health care services used per week. In the Stability and Order phase, there were two significant spikes in recording the refugees by health care providers with respective changes in the number of identified medical conditions and increases of the number of used of health care services. As previously described, they were probably the result of the joint activity of international, humanitarian agencies and domestic public and private health care providers [42]. The quotas and tensions phase was marked by a decrease in health service utilization. This was probably a reflection of changes in the health care seeking behavior in response to the political decision to set hard quotas of acceptance to the EU [5].

This study results highlight the size of the effects of regional and national political response to the health demands of refugees which might be considered in planning the efforts for the provision of health care in both transit and destination countries in the future refugee crises [10]. In congruency, a policy implication was the direct and positive relationship between the number of arrivals of refugees in Serbia and the number of medical conditions identified, as well as the number of health care services used. This fact strongly advocates for the use of modelling techniques in planning the response of health care capacities for accommodating arrivals of refugees shaped by the political decisions. Finally, this study has established ARIMA models as a resource contributing to a better understanding of the provision of health care for refugees in a potential crisis. ARIMA models are an effective representation of the influence of changes in political context affecting the number of refugee arrivals to Serbia and EU, as well as the numbers of registered refugees by health care providers, of identified medical conditions and of the health care services used.

Several contextual factors drive national contingency planning for responding to the influx of migrants, refugees, and asylum-seekers, including national health strategic planning. According to guidance from the WHO Europe Region [3], evaluating health system readiness to respond to such a crisis involves assessing the explicit consideration of this population in government planning for the health sector. Ensuring the provision of primary health care with adequately-trained staff, efficient use of resources, and procedures for screening, case finding, and triage of patients should be explicit [43].

Optimizing the ability of the health sector to respond effectively may also be evaluated by guidance from IOM and UNHCR’s Project PHAME that aimed to support national and subnational authorities in leading multi-sectoral preparation for large-scale influxes of transient at-risk populations. The gap analysis purported by this project aims at assisting health authorities in building on existing national capacities and developing informed interventions for arrivals of large groups of migrants [44].

This study has been affected by a number of policy implications. First, in March 2018, the Assembly of the Republic of Serbia adopted the new Law on Asylum and Temporary Protection, which among other novelties accelerates procedures for asylum-seeking, border crossings and for defining the list of safe countries, and establishes individualized, gender-based approach in transit zones [34]. The study limitations derive from the accuracy of original data and scarcity of available data on refugees. Accounting for errors in initial data collection, an underestimation of study finding is more likely than an overestimation, since it focused on legal migrations only and their health needs that were met by the public health care system. There is no precise information about illegal migrations and unmet needs of the refugees. These aspects need to be further explored, and more knowledge is needed on the correlation of demographic data with health status and demand for health care services among refugees in transition countries. In addition, due to the lack of other data (such as age, sex, nationality, education level, health history and health care coverage), this study provides only a broad picture of the effect of political decisions on migration flows and on the capacity of national health system to respond to the health needs of refugees transiting Serbia. The paper does not study differences between regions. A limitation to assessing how Serbia’s public healthcare system responded to the crisis is an evidence gap in the quality of care received by migrants, as an assessor of the quality of health systems. In the future, it is necessary for Serbia and other countries in the region to ensure contingency planning for the migrant influx is part of the national health planning agenda to be effectively-prepared to handle future migrant crises.

Serbia’s health system showed the ability of primary healthcare providers to adapt treatment guidelines efficiently to provide quality screening and care to migrants. The study methods have helped to specify general models of five time-series parameters and based on the information of the external factors such as political changes whose joint impact was estimated on the refugee week arrivals in Serbia and to the EU, as well as the weekly number of health care service used and medical conditions of refugees in each period of the WBRC evolution. In the future, ARIMA models can be used in all other crises to immediately detect causes of the changes and whether some political decisions have had an impact or not. If a political decision has an impact on crises, it will be an event in time series modelling and will be recognized as an outlier. The outlier can be further used as an intervention variable to perform the ARIMA model without shocks, for instance, to define the net impact of political changes on the observed time series model.

## 5. Conclusions

Serbia, a transit country during the 2015/2016 WBRC, coped efficiently with the influx of refugees. With considerable international technical and financial assistance, the country provided increased health care coverage to this vulnerable population. Health care providers timely treated all acute health conditions and addressed the needs of refugees without straining health care services. The study provided evidence that political decisions rapidly shaped the number of arrivals of refugees in Serbia and to EU and provides valuable lessons on policy options for addressing humanitarian crises in transit and destination countries. Thus, this study informs the prediction of the flows of refugees, their health needs, and demands for services in correspondance to political decsion making, in the case of repeat refugee crises in the future.

## Figures and Tables

**Table 1:** Weekly reported number, medical conditions, and health care services used by refugees in Serbia during the Western Balkan Refugee Crises 2015/2016.

Number	Mean ± Standard Deviation (Minimum and Maximum Number)				
	Prior to August 2015 & August–September: Chaos and Panic (sum of Period I and II, 1 June–4 October 2015)	October–November: Stability and Order (Period III, 5 October–22 November 2015)	November–February: Restrictions and Confusion (Period IV, 23 November 2015–14 February 2016)	Mid-February: Quotas and Tensions (Period V, 15 February–6 March 2016)	March: EU—Turkey Summit Meeting (Period VI, 7 March–3 April 2016)
Arrivals in Serbia	…	43,051.3 ± 9029.9 (32,250–54,691)	16,592.1 ± 4365.4 (9209–23,574)	2861.7 ± 1457.9 (1454–4365)	100.5 ± 67.9 (30–184)
Arrivals to EU	…	137,198.0 ± 23,598.9 (101,901–16,1405)	55,762.1 ± 15,299.3 (31,095–80,358)	9323.3 ± 3821.4 (6866–13,726)	1727.8 ± 217.9 (1502–2023)
Registered by health care providers	1734 ± 957.6 (276–2583)	5021.1 ± 3420.9 (1785–10,833)	3565.9 ± 658.9 (2482–4474)	2641.7 ± 512.9 (2249–3222)	2139.0 ± 2171.0 (1651–2563)
Medical conditions	1733.0 ± 947.3 (301–2603)	4882.6 ± 3412.1 (1811–10,856)	3578.6 ± 653.4 (2484–4502)	2633.0 ± 511.3 (2261–3216)	2105.0 ± 364.5 (1646–2501)
Health care services used	1958.0 ± 856.3 (705–2769)	5091.6 ± 3424.7 (1900–10,952)	3679.1 ± 690.1 (2559 –4546)	2676.0 ± 470.8 (2369–3218)	2697.3 ± 1116.6 (1790–4322)
Check-up of persons seeking asylum	38.8 ± 42.6 (1–100)	2.4 ± 3.6 (0–9)	115.3 ± 337.3 (0–1179)	9.3 ± 16.2 (0–28)	23.8 ± 5.9 (18–32)

**Table 2:** Statistical parameters of the Autoregressive Integrated Moving Average (ARIMA) models of number of refugees’ arrivals in Serbia (Model A) and in the EU (Model B), registered in healthcare institutions (Model C), their medical conditions (Model D), and health care services used (Model E) during the Western Balkan Refugee Crisis 2015/2016.

Model	Week Number: Type of Outliers	Implicit and Explicit Model Specification	Ljung-Box Q (18) Fit Statistics
A ARIMA (0,1,0)	w 11: Additive	$\left(1-B\right)Y_{t}=\varepsilon_{t}+\omega \cdot I_{Week11}\Rightarrow Y_{t}=Y_{t-1}+19,713.998\cdot I_{Week11}+\varepsilon_{t}$	11.477 R^2^ = 0.415 *p* = 0.873
B ARIMA (0,1,0)	w 6: Innovational; w 8: Transient; w 11: Additive; w 24: Additive	$\left(1-B\right)Y_{t}=\theta_{0}+\varepsilon_{t}+\omega_{1}\cdot I_{Week6}+\omega_{2}\cdot \frac{1}{1-\delta \cdot B}\cdot I_{Week8}+\omega_{3}\cdot I_{Week11}+\omega_{4}\cdot I_{Week24}\Rightarrow$ $Y_{t}=Y_{t-1}-5,317.357+78,305.349\cdot I_{Week6}+48,681.588\cdot \frac{1}{1-0.793\cdot B}\cdot I_{Week8}+$ $+45,508.943\cdot I_{Week11}+29,865.777\cdot I_{Week24}+\varepsilon_{t}$	14.311 R^2^ = 0.810 *p* = 0.709
C ARIMA (0,0,0)	w 1 Transient w 9 Additive; w 11 Additive; w 25 Local Trend	$Y_{t}=\theta_{0}+\varepsilon_{t}+\omega_{1}\cdot \frac{1}{1-\delta \cdot B}\cdot I_{Week1}+\omega_{2}\cdot I_{Week9}+\omega_{3}\cdot I_{Week11}+\omega_{4}\cdot \frac{1}{{\left(1-B\right)}^{2}}\cdot I_{Week25}\Rightarrow$ $Y_{t}=3,607.528-5,317.357\cdot \frac{1}{1-0.768\cdot B}\cdot I_{Week1}+7,603.479\cdot I_{Week9}+$ $+5,056.424\cdot I_{Week11}-283.710\cdot \frac{1}{{\left(1-B\right)}^{2}}\cdot I_{Week25}+\varepsilon_{t}$	24.783 R^2^ = 0.899 *p* = 0.131
D ARIMA (0,0,0)	w 9 Additive; w 10 Transient; w11 Additive	$Y_{t}=\theta_{0}+\varepsilon_{t}+\omega_{1}\cdot I_{Week9}+\omega_{2}\cdot \frac{1}{1-\delta \cdot B}\cdot I_{Week10}+\omega_{3}\cdot I_{Week11}\Rightarrow$ $Y_{t}=31,821.324+9,034.676\cdot I_{Week9}+2,624.214\cdot \frac{1}{1-0.941\cdot B}\cdot I_{Week10}+$ $+3,755.860\cdot I_{Week11}+\varepsilon_{t}$	16.659 R^2^ = 0.883 *p* = 0.547
E ARIMA (0,0,0)	w1 Transient; w 9 Additive; w 11 Additive	$Y_{t}=\theta_{0}+\varepsilon_{t}+\omega_{1}\cdot \frac{1}{1-\delta \cdot B}\cdot I_{Week1}+\omega_{2}\cdot I_{Week9}+\omega_{3}\cdot I_{Week11}\Rightarrow$ $Y_{t}=3,365.052-2,610.845\cdot \frac{1}{1-0.707\cdot B}\cdot I_{Week1}+7,750.271\cdot I_{Week9}+$ $+5,246.617\cdot I_{Week11}+\varepsilon_{t}$	25.571 R^2^ = 0.829 *p* = 0.110

Note: Dummy variable I_Week_ is binary type, and is equal zero, except in concrete week, when takes value one.

## Supplementary Materials

The following are available online at https://www.mdpi.com/2227-9032/8/4/560/s1. Table S1: ARIMA with time series modeling outliers.
