# Supplementary material for: Uptake of Health Care Services by Refugees: Modelling a Country Response to a Western Balkan Refugee Crisis
Source: Healthcare (Basel). 2020 Dec 14;8(4):560. doi: 10.3390/healthcare8040560 (PMC7765010; doi:10.3390/healthcare8040560)
Supplement: Supplementary file 1 [file healthcare-08-00560-s001.pdf]

Supplemental file 1: ARIMA with time series modeling outliers

ARIMA tme series Model	Political decisions	Outliers					
		Week	Type	Estimate	SE	t	Sig.
A. Refugees' arrivals in Serbia, n	**25 Oct 2015 (III period - Stability and Order):** Meeting on the Western Balkans Migration Route -17 point action plan;	**11** (9-11 Nov 2015)	Additive (spike/shock, sudden change)	19713.998			0.000
B. Refugee's arrivals to the EU countries, n	**14 Sept 2015 (II period - Chaos and Panic):** Extraordinary Justice and Home Affairs Council.<br>**22 Sept 2015:**<br>Extraordinary Justice and Home Affairs Council;<br>**23 Sept 2015:**<br>Extraordinary EU Council meeting	**6** (5 - 11 Oct 2015)	Innovational (slow change in the process continues forward through time)	78305.349	13235.784	5.916	0.000
	**8.10.2015 (III period - Stability and Order)**: High-level Conference on the Eastern Mediterranean - Western Balkans route;<br>**15.10.2015 (III period - Stability and Order):** European Council - EU leaders agreement	(19 - 25 [illegible]ct 2015)	Transient Magnitude (spike/shock that gradually disappear)	48681.588	12621.248	3.857	0.001
			Decay factor	0.793	0.213	3.731	0.001
	**25 Oct 2015 (III period - Stability and Order):** Meeting on the Western Balkans Migration Route – 17 point action plan;	**11** (9-11 Nov 2015)	Additive	45508.943	9228.553	4.931	0.000
	**Jan 2016 (IV period – Restrictions and Confusion):** Austria and Germany cap the refugee influx, plan repatriation and deportation of criminal refugees; Austria stricter border controls and new fences along the Slovenian border;<br>**1 – 8 Feb 2016 (IV period – Restrictions and Confusion):** Macedonia shuts the borders, constructs a second fence with Greece	**24** (8 – 14 Feb 2015)	Additive	29865.777	9177.509	3.254	0.004
C. Refugees registered in healthcare institutions, n	**May (I period – Prior to August);** A European Agenda on Migration;<br>**Sept (II period - Chaos and Panic):** European Centre of Diseases Control issued advice on the public health needs of migrants across Europe Union.	**1** (1 June – 6 Sept 2015)	Transient Magnitude	-3123.573	577.752	-5.406	0.000
			Decay factor	0.768	0.079	9.758	0.000

	**25 Oct 2015 (III period - Stability and Order):** Meeting on the Western Balkans Migration Route – 17 point action plan;	**9** (26 Oct - 1 Nov 2015)	Additive	7603.479	728.273	10.440	0.000
		**11** (9 - 11 Nov 2015)	Additive	5056.424	718.488	7.038	0.000
	**15 Feb 2016 (V period - Quotas and tensions)**: Visegrad group closure of the Balkan route, Austria expands the list of safe countries of origin;<br>**19 Feb 2016:** Austria and Germany cap number of asylum applications per day + persons allowed to transit per day;<br>**21 Feb 2016:** The North Macedonia passes new restrictions, Serbia, Croatia and Slovenia reduce their numbers of refugees;	**25** (15 – 21 Feb 2015)	Local Trend	-283.710	72.714	-3.902	0.001
D. Medical conditions of refugees in Serbia, n	**25 Oct 2015 (III period - Stability and Order):** Meeting on the Western Balkans Migration Route – 17 point action plan;	**9** (26 Oct – 1 Nov 2015)	Transient Magnitude	9034.676	768.194	11.761	0.000
			Decay factor	2624.214	483.829	5.424	0.000
		**10** (2 - 8 Nov 2015)	Additive	0.941	0.020	46.059	0.000
		**11** (9 - 11 Nov 2015)	Additive	3755.860	813.743	4.616	0.000
E. Health care services used by refugees in Serbia, n	**May (I period – Prior to August):** A European Agenda on Migration;<br>**Sept:** European Centre of Diseases Control issued advice on the public health needs of migrants across Europe Union.	**1** (1 June – 6 Sep 2015)	Additive	-2610.845	773.766	-3.374	0.002
	**25 Oct 2015 (III period - Stability and Order):** Meeting on the Western Balkans Migration Route – 17 point action plan;<br>Personnel Health Record for migrants is launched;	**9** (26 Oct - 1 Nov 2015)	Transient Magnitude	0.707	0.138	5.143	0.000
			Decay factor	7750.271	904.878	8.565	0.000
		**11** (9 - 11 Nov 2015)	Additive	5246.617	896.648	5.851	0.000
